# Supplementary material for: Comprehensive Assessment of Water Quality and Pollution Source Apportionment in Wuliangsuhai Lake, Inner Mongolia, China
Source: Int J Environ Res Public Health. 2020 Jul 14;17(14):5054. doi: 10.3390/ijerph17145054 (PMC7399887; doi:10.3390/ijerph17145054)
Supplement: Supplementary file 1 [file ijerph-17-05054-s001.pdf]

Supplementary Information

Comprehensive assessment of water quality and pollution source apportionment in Wuliangsuhai Lake, Inner Mongolia, China

Rui Shi [1,2], Jixin Zhao [3], Wei Shi [4], Shuai Song [1,5]*, and Chenchen Wang[1,5]

1 State Key Laboratory of Urban and Regional Ecology, Research Center for Eco-Environmental Sciences, Chinese Academy of Sciences, Beijing 100085, China; s18247808609@163.com(R.S.) shuaisong@rcees.ac.cn(S.S.); wccapp@126.com(C.W)
2 Environmental Information Monitoring Center of Bayannur, Bayannur 015000, China; s18247808609@163.com(R.S.)
3 Institute of Environmental Science of Bayannur, Bayannur 015000, China; zhaojixin1984@163.com(J.Z.)
4 Institute of Loess Plateau, Shanxi University, Taiyuan 030006, China; shiwei@sxu.edu.cn(W.S.)
5 University of Chinese Academy of Sciences, Beijing 100049, China
* Correspondence: shuaisong@rcees.ac.cn; Tel.: +86-010-62844160

Received: date; Accepted: date; Published: date

Table S1. Nonparametric test for the distribution of water-quality factors in Wuliangsuhai Lake

Item	Sampling position			Sampling period		
	Chi-square value	Degree of freedom	sig.	Chi-square value	Degree of freedom	sig.
pH	37.011	18	0.005	25.99	5	0.01
Turbidity	29.226	18	0.046	27.915	5	0.001
TSS	50.676	18	0.001	10.175	5	0.07
Salinity	43.512	18	0.001	32.1	5	0.001
Transparency	46.867	18	0.001	22.268	5	0.001
Chlorophyll a	24.16	18	0.15	53.634	5	0.001
DO	25.102	18	0.122	26.18	5	0.001
$KMnO_4$	43.731	18	0.001	26.444	5	0.001
BOD	41.674	18	0.001	20.266	5	0.001
COD_{Mn}	32.207	18	0.021	41.607	5	0.001
TN	29.467	18	0.043	8.187	5	0.146
NH_3-N	18.831	18	0.402	8.09	5	0.151
TP	36.427	18	0.006	16.308	5	0.006
Fluoride	43.589	18	0.001	44.072	5	0.001
As	8.749	18	0.965	75.105	5	0.001
Hg	6.256	18	0.995	79.319	5	0.001
Pb	14.441	18	0.7	38.189	5	0.001
Cu	9.147	18	0.956	52.157	5	0.001
Zn	21.943	18	0.235	44.198	5	0.001
Cd	14.272	18	0.711	35.327	5	0.001
Se	7.762	18	0.982	50.887	5	0.001
Coliform bacteria	62.753	18	0.654	3.735	5	0.588

Table S2. P values for post hoc pair-wise comparisons of indicators for sampling sites using the Conover-Iman test*

	pH	Turbidity	TSS	Salinity	Transparency	$KMnO_4$	BOD	COD_{Mn}	TN	TP
**Turbidity**	**<0.0001**									
**TSS**	**<0.0001**	**0.0056**								
**Salinity**	**<0.0001**	**<0.0001**	**<0.0001**							
**Transparency**	**<0.0001**	**<0.0001**	**<0.0001**	**<0.0001**						
**$KMnO_4$**	**0.0445**	**<0.0001**	**<0.0001**	**<0.0001**	**<0.0001**					
**BOD**	**<0.0001**	**<0.0001**	**<0.0001**	**<0.0001**	**<0.0001**					
**COD_{Mn}**	**<0.0001**	**<0.0001**	**<0.0001**	**<0.0001**	**<0.0001**	**<0.0001**	**<0.0001**			
**TN**	**<0.0001**	**<0.0001**	**<0.0001**	**<0.0001**	**<0.0001**	**<0.0001**	**<0.0001**	**<0.0001**		
**TP**	**<0.0001**	**<0.0001**	**<0.0001**	**<0.0001**	**<0.0001**	**<0.0001**	**<0.0001**	**<0.0001**	**<0.0001**	
**Fluoride**	**<0.0001**	**<0.0001**	**<0.0001**	**<0.0001**	**<0.0001**	**<0.0001**	**<0.0001**	**<0.0001**	**<0.0001**	**<0.0001**

* Bold indicates significance ($P \leq 0.05$)

Table S3. Total variances explained by PCA

Component	irrigation period					
	Initial eigenvalues			Extracting square and loading		
	Total	Variance %	Accumulation %	Total	Variance %	Accumulation %
1	3.329	15.13	15.13	3.329	15.13	15.13
2	2.543	11.558	26.688	2.543	11.558	26.688
3	2.256	10.256	36.945	2.256	10.256	36.945
4	1.889	8.588	45.533	1.889	8.588	45.533
5	1.652	7.507	53.04	1.652	7.507	53.04
6	1.304	5.928	58.968	1.304	5.928	58.968
7	1.259	5.724	64.692	1.259	5.724	64.692
8	1.037	4.712	69.404	1.037	4.712	69.404
9	0.979	4.449	73.853			
10	0.916	4.161	78.015			
11	0.705	3.206	81.221			
12	0.65	2.956	84.177			
13	0.549	2.494	86.671			
14	0.497	2.258	88.929			
15	0.461	2.095	91.024			
16	0.437	1.984	93.008			
17	0.376	1.71	94.718			
18	0.31	1.411	96.128			
19	0.292	1.329	97.457			
20	0.264	1.202	98.659			
21	0.194	0.88	99.54			
22	0.101	0.46	100			

Table S4. The rotated PCA component matrix

Component	1	2	3	4	5	6	7	8
pH	-0.111	0.065	**-0.734**	0.128	-0.178	-0.087	-0.097	0.002
Turbidity	0.127	**0.715**	-0.17	0.186	-0.323	-0.009	0.021	0.224
TSS	0.385	**0.653**	-0.164	-0.061	0.163	0.096	0.064	-0.19
Salinity	**0.643**	-0.09	0.461	0.109	-0.119	0.281	0.016	0.063
Transparency	-0.159	**-0.756**	-0.171	-0.065	0.237	-0.245	0.181	-0.016
Chlorophyll a	-0.058	0.16	-0.092	0.136	0.015	0.033	0.087	**0.862**
DO	0.167	-0.035	-0.006	0.28	0.76	0.045	-0.176	0.201
$KMnO_4$	0.44	0.116	-0.059	0.183	**-0.692**	-0.127	-0.118	0.093
BOD	**0.909**	0.075	0.149	-0.037	0.074	-0.04	0.059	-0.017
COD_{Mn}	**0.887**	0.193	-0.044	0.073	-0.129	-0.098	0.041	-0.056
TN	-0.025	0.119	0.097	0.032	0.422	**0.597**	0.111	-0.085
NH_3-N	-0.023	-0.063	-0.034	-0.061	-0.038	**0.858**	-0.069	0.023
TP	-0.23	**0.647**	0.05	-0.187	0.311	-0.058	0.068	0.188
Fluoride	0.331	-0.004	0.061	-0.078	-0.359	-0.024	**0.558**	0.152
As	0.196	-0.079	0.178	**0.702**	0.134	-0.217	-0.025	0.151
Hg	0.07	-0.11	0.187	**-0.784**	0.015	-0.023	-0.097	-0.018
Pb	-0.077	-0.109	-0.078	0.493	0.014	0.016	**0.572**	-0.342
Cu	0.11	-0.077	-0.184	0.226	0.03	0.102	**0.715**	0.094
Zn	0.125	-0.025	**0.629**	-0.061	-0.03	0.179	-0.355	-0.06
Cd	-0.041	0.106	**0.601**	0.158	-0.214	-0.111	0.09	-0.481
Se	0.137	-0.151	-0.241	0.34	-0.065	0.143	**-0.564**	0.036
Coliform bacteria	0.009	0.29	0.287	-0.096	0.039	0.606	-0.001	0.127

Table S5. The PCA component score

	PC1	PC2	PC3	PC4	PC5	PC6	PC7	PC8
	0.37642	1.81152	1.16549	0.14203	-0.54335	0.81239	1.08099	-0.33862
	0.62959	-0.31802	2.02408	0.17059	-0.06729	2.51464	0.72875	0.17515
	0.24269	2.85946	0.22735	0.68361	0.84534	-0.12568	0.46114	-0.1566
	0.82702	-0.40001	1.32097	0.25738	-0.29954	0.20916	0.56812	-0.00833
	-0.36237	0.15645	0.30938	0.56201	1.63301	-0.81107	0.17329	0.75075
	-0.43132	-0.8534	-0.26839	0.99229	2.17439	0.44034	0.55145	-0.24894
	-0.07591	-1.15149	-0.61378	0.4049	0.24225	-0.42546	0.30508	1.13479
	0.22612	0.55371	-0.05239	0.47273	0.4475	-0.30922	0.24076	0.30219
	0.48712	-0.08769	-0.74904	1.3447	0.2149	-0.08134	0.75359	0.66922
	0.03407	0.34467	0.32239	1.05381	0.30926	-0.49608	1.25892	-1.24272
	-0.65712	-0.8194	0.02424	1.70791	0.30234	1.26821	3.18652	-1.4573
	-0.23094	-1.0813	0.23473	0.38439	-0.25629	-0.58908	0.25994	1.19716
	0.11063	-0.91923	0.28999	0.89502	0.2993	-0.36119	1.23235	0.60781
	-0.1892	-0.93197	-0.10586	0.8125	0.31411	-0.75807	1.26637	0.40255
	-0.09507	-1.34733	0.27613	0.43554	0.30315	-0.26371	0.81566	0.55728
	0.31799	-1.20131	0.01562	1.61745	1.42384	0.91289	2.96807	-0.03236
	0.54486	-1.88044	0.12785	1.46879	0.25053	0.67326	3.66536	-1.36205
	-0.03295	-0.86052	0.31434	1.93021	0.05796	-0.90781	0.34755	0.44471
	1.0825	-0.79884	-0.84509	0.71527	0.53058	-0.7399	0.41424	0.23057
	0.93219	0.70011	0.31932	-0.2194	0.6767	0.42822	-0.97676	-0.03947
Nonirrigation	4.42645	-0.08859	0.79253	-1.08077	2.03804	0.11262	-0.95006	-0.22104
	1.435	1.69881	-1.04071	-0.89486	1.9967	0.58394	-0.34176	-1.02298
	1.09835	-0.17277	1.68354	1.90007	0.63216	0.8794	-0.29134	0.42929
	0.98846	-1.30237	0.47039	0.38576	1.60607	-1.12613	-0.24224	0.64053
	-0.12665	-1.70978	-0.03326	-0.39483	0.77216	-0.41284	-1.02297	0.11942
	0.09219	-0.41333	-0.37276	-0.23541	0.31781	-0.49588	-0.49702	1.41342
	0.02241	0.38601	-0.03647	-0.67541	0.42558	-0.65907	-0.22405	0.59053
	0.26767	0.04097	-0.72137	-0.08371	-0.42218	-0.96069	-0.61647	0.99419
	-0.26966	0.14261	-0.17528	-0.36725	0.46277	0.14249	-0.23433	0.08827
	0.7404	-0.67316	0.13293	-0.52163	0.19353	-0.72576	-0.65041	-0.56239
	1.45711	-0.42348	-1.22855	-0.237	0.53786	-1.0294	-0.98832	0.84628
	0.31649	-0.9407	-0.34002	-0.44055	0.61312	-0.89104	-1.08373	1.11269
	0.68987	-2.53201	-2.69549	1.69963	-0.24175	1.57186	-3.90828	-0.07715
	0.19032	-1.5817	-0.3531	0.1394	0.12591	0.07309	-0.82606	1.01595
	0.15112	0.51053	-0.25315	-0.65143	0.92166	-0.83089	-0.54766	0.68033
	0.80073	-1.09243	0.22169	-0.34964	-0.59278	-0.43881	-0.43066	-0.75176
	0.00395	-0.77536	-0.0444	-0.05699	-0.09927	0.65585	-0.88609	0.07471
	1.18273	-0.12493	1.03956	0.17372	-0.31018	-0.74537	-0.41223	-1.81227
	-0.33538	-0.06661	-0.68916	2.26399	-0.20081	0.16999	-2.78682	-0.55137
	1.62414	-0.88014	3.80485	0.56168	-0.11696	0.38022	-2.22745	-0.40708
	-0.67624	1.72854	0.3205	0.83553	1.89245	0.8072	-1.04759	-1.43857

	0.91692	0.73002	2.13441	1.52936	-1.39784	-0.07069	-1.70068	-0.067
	-1.38	-0.61222	-0.82235	0.44337	0.72146	-0.54478	-0.81748	-0.60036
	-0.61447	-1.06141	-0.96078	0.39666	0.75908	-0.58572	-0.74172	-0.30663
	-1.42841	0.7288	-0.00057	3.82845	-0.22385	-0.15926	1.42016	-3.14601
	-0.5096	1.0668	0.26567	1.37649	-0.56702	-0.68065	-0.89379	-0.31113
	-0.23476	0.97151	0.015	1.68159	-0.61536	-0.97535	-1.29753	-0.78706
	0.03168	-0.25413	-0.10027	2.2843	0.37034	-0.42952	-0.86087	0.62321
	-0.20049	0.43227	0.07568	1.53583	-0.71472	-0.55218	-0.86721	-0.41704
	0.06852	1.31186	-0.08822	1.08427	-0.91068	-0.41669	-1.03953	-0.6743
	-0.17554	0.25491	0.28467	0.89955	-1.32484	-0.56195	0.053	0.17543
	-0.29055	1.30714	1.21029	1.18462	-0.94338	-0.70765	0.04642	0.46776
	0.06766	0.65123	0.64378	0.88101	-1.35616	-0.05231	-0.22337	0.08606
	0.69557	0.87402	0.8408	1.01851	-0.40889	-0.99103	-0.01409	0.24836
	5.35018	-0.17936	-0.89434	-1.26443	1.3322	-0.22912	1.2039	-0.63888
	-0.12442	1.08453	-0.37339	1.17763	0.04634	0.42524	-0.6207	-0.34737
	-0.56155	0.26851	0.83072	0.37106	-0.53227	-0.30099	-0.25005	-0.24857
	-0.74698	1.31224	-0.61382	-0.13553	-0.2359	0.10034	0.94039	1.08898
	1.73317	-0.90618	0.61761	-0.23845	0.41302	1.42037	0.45326	0.24992
	-0.49984	2.78847	-0.30691	-0.23641	1.23689	1.38431	0.10997	-0.44988
	0.71403	0.50044	0.01249	-0.87216	-2.05381	0.53139	0.34114	0.61801
	-1.10228	-1.1585	-2.11834	-0.20461	-0.09297	0.26785	0.9102	-1.02879
	-0.6514	-1.20961	-0.11727	-0.98834	0.78659	-0.31886	0.65559	-0.03659
	0.60809	0.16026	-1.94092	-0.84108	-0.9792	-0.47492	0.14265	-0.39214
	0.23847	0.91492	-1.3216	-0.51066	-1.26195	-0.59765	0.30818	0.16385
	0.25848	0.22435	-1.17288	-0.2839	-1.3407	-0.13701	0.8364	0.29835
	-0.85178	-1.03517	-1.22941	-0.4468	-0.75592	0.0476	1.01815	-0.72053
	-0.01301	-0.61812	-1.49377	-0.49231	-1.40176	0.10565	0.00289	-0.36763
	0.79007	0.64903	-1.66146	-0.12783	-1.39894	-0.16004	1.3362	-0.94113
	0.34645	-0.66941	-1.29129	-0.39935	-1.26306	-0.08593	0.15357	-0.76253
	0.60597	-0.439	-0.72318	-0.3934	-0.9839	0.63473	0.93316	-0.56375
	1.06868	0.14626	-0.14416	-1.40713	-1.50489	-0.00187	0.91291	-0.19519
	0.62255	0.1983	-0.28287	-0.52097	-1.62533	-0.07076	0.05276	0.05872
	1.09694	0.1027	0.29276	-1.27161	-1.99577	-0.17615	1.53842	-0.0498
	0.86326	2.29432	-1.64701	-0.45919	-1.13134	0.11116	0.02454	-0.32306
	1.09288	1.14046	-0.81583	-0.43762	-2.16013	0.1765	0.06186	-1.03505
	-0.53066	2.06758	0.66986	0.76128	1.21031	0.33785	1.96313	6.41688
	-0.52482	-0.54588	-1.5592	0.03704	0.84647	6.19106	-0.61913	0.41399
	-0.31413	4.15609	-1.98711	-0.91444	2.39757	0.37473	-0.4355	-1.50367
	0.38235	0.69807	0.40229	0.24443	-2.51118	-0.03809	-0.04156	0.99175
irrigation	-1.21225	-0.75611	-0.61745	-0.67605	0.76308	0.45668	-0.15073	0.37099
	-1.33739	-0.29184	-0.76296	-0.36243	0.56261	-0.34649	-0.48649	-0.05058
	-1.06544	-0.24607	-0.66512	-0.73319	0.8486	0.02872	-0.27694	0.53361
	-0.93975	0.84853	-0.25735	0.09406	0.5656	-0.92509	0.66114	1.17528
	-0.29668	-0.06543	-0.78153	0.00673	-0.11746	-0.67625	-0.70731	0.50374

-1.03856	-0.08523	-0.9565	-0.06814	0.27971	0.84618	-0.49903	1.91673
-1.1595	-0.19726	-1.03137	-0.429	-0.37491	-0.17695	-0.55788	0.75729
-0.41792	0.06199	-0.65596	0.31616	0.25602	0.25851	-0.73848	0.91185
-0.82472	-0.30386	0.00479	-0.13093	-0.12835	1.04562	-0.0727	0.71078
-0.52757	0.50614	-0.38606	-0.26884	-0.87173	-0.01986	-0.25355	1.54915
-0.25994	0.18414	0.53066	-0.2283	-1.33993	-0.04218	0.41737	1.34481
0.23679	0.22833	0.21236	-0.56717	-0.43086	-0.85823	0.56762	1.11245
0.58872	0.14468	-0.11912	-0.06232	-0.99386	-0.16285	-0.3009	0.90632
-0.53591	-0.25349	-0.57436	-1.27304	0.41821	0.63722	0.34274	0.16432
-0.06897	-0.23257	-0.41846	-1.03325	-0.1863	-0.17591	0.09954	-0.23492
-1.10776	0.28012	2.69857	-1.11667	-0.94426	0.58987	-0.83196	0.54726
-0.67617	-0.45289	1.90834	-1.24161	-1.91332	4.83266	-0.77482	0.03295
0.12283	1.14351	1.49583	-1.19758	2.62443	1.26406	0.01973	-0.65213
0.23284	-0.55225	0.53223	-0.86701	0.06997	-0.35606	0.01851	-0.47735
-1.24619	-0.314	1.26544	-1.0645	0.77821	-1.00072	0.15879	-1.06713
-1.00024	0.05614	0.58189	-0.9995	1.10153	0.00802	-0.18572	-0.47445
-1.27414	-0.14941	1.04756	-2.25962	0.62027	-0.92503	0.18531	-0.73916
-0.47159	-0.09833	0.8181	-0.91849	0.1925	-0.61718	-0.19054	-0.54995
-1.04812	-0.34551	1.05473	-1.1107	0.50157	-1.01299	0.39581	-0.77012
-1.45957	0.11027	0.77483	-0.37047	-0.1146	-0.15332	0.06363	-1.06496
-1.05528	-0.15344	0.58225	-0.59831	1.2247	-0.9567	-0.49606	-0.47631
-1.32017	-0.01051	-0.4351	-0.91304	-1.17352	0.9789	-0.34272	-1.00584
-1.18539	-0.53325	0.62743	-1.16597	-0.16069	-0.66043	-0.27828	-0.70953
-0.50276	-0.27943	0.00087	-1.32477	0.07614	-0.73849	-0.2933	-0.65861
-0.43885	-0.69046	0.66505	-0.67792	-0.04992	-0.50573	0.01097	-0.64791
-1.01065	-0.64082	1.92578	-1.02787	-0.0917	-0.83121	-0.08584	-0.7614
-0.47227	-0.09776	0.372	-1.48235	0.28556	-0.89449	0.17427	-0.09396
-0.97649	-0.46112	1.01196	-1.42827	-0.55925	0.2377	0.77272	-0.04522
-0.86398	-0.20403	0.10197	-0.91095	0.44283	-0.38883	-0.44637	-0.79201

Table S6. Analysis results of all samples.

Date	Site	T(℃)	pH	Turbidity	TSS	Salinity	Transparency （cm）	Chlorophyll a (mg/L)	DO	KMnO4	BOD	CODMn	TN	NH3-N	TP	Oil	Fluoride	Anionic surfactants	As	Hg	Pb	Cu	Zn	Cd	Se	Coliform bacteria （num/L）
2015-6-8	1	20.0	8.05	31	56	2916	45	0.01734	4.5	7.9	4.1	45	2.110	0.184	0.081	0.039	0.72	0	0.00205	0.000021	0.0021	0.0063	0.043	0.00047	0.00000	170
2015-6-8	2	21.0	8.11	6	11	5824	55	0.00876	5.4	6.6	3.9	40	3.380	0.299	0.081	0.026	0.69	0.083	0.00151	0.000019	0.0015	0.0040	0.047	0.00034	0.00010	230
2015-6-8	3	20.0	8.43	33	63	2260	30	0.01268	6.3	7.3	4.0	42	1.570	0.089	0.165	0	0.62	0.133	0.00431	0.000023	0.0026	0.0064	0.007	0.00029	0.00019	170
2015-6-8	4	21.0	8.11	5	16	3356	50	0.01136	6.1	8.1	4.4	51	1.640	0.096	0.032	0.049	0.74	0	0.00260	0.000022	0.0016	0.0039	0.019	0.00039	0.00000	110
2015-6-9	5	19.6	8.47	6	8	1924	105	0.00989	8.3	7.3	2.7	33	1.720	0.105	0.165	0	0.62	0	0.00471	0.000026	0.0025	0.0038	0.000	0.00011	0.00007	50
2015-6-9	6	19.6	8.49	3	9	1892	165	0.00680	9.0	6.5	2.3	28	3.260	0.290	0.112	0	0.60	0	0.00379	0.000020	0.0093	0.0028	0.000	0.00014	0.00008	20
2015-6-9	7	19.8	8.54	5	18	1788	150	0.03358	5.8	7.8	2.7	35	1.680	0.100	0.049	0	0.66	0	0.00367	0.000021	0.0037	0.0028	0.000	0.00000	0.00018	2
2015-6-9	8	19.9	8.50	23	26	1744	90	0.02429	6.8	7.2	4.1	44	2.010	0.124	0.077	0	0.68	0	0.00312	0.000024	0.0022	0.0025	0.000	0.00033	0.00018	50
2015-6-9	9	19.8	8.53	25	26	1704	100	0.03097	8.2	9.7	3.1	48	1.800	0.192	0.039	0	0.72	0	0.00348	0.000021	0.0119	0.0053	0.007	0.00011	0.00013	2
2015-6-9	10	20.4	8.37	14	30	1846	80	0.01210	5.4	6.3	4.1	46	1.630	0.096	0.063	0	0.64	0	0.00374	0.000023	0.0152	0.0048	0.006	0.00043	0.00000	20
2015-6-9	11	20.8	8.14	7	16	1896	120	0.01101	4.2	6.6	2.5	36	3.340	0.297	0.063	0	0.60	0	0.00352	0.000022	0.0258	0.0183	0.000	0.00031	0.00011	2
2015-6-9	12	20.1	8.38	6	5	1808	120	0.02667	6.4	8.7	2.7	33	0.999	0.057	0.047	0	0.72	0	0.00362	0.000028	0.0050	0.0015	0.000	0.00011	0.00016	70
2015-6-9	13	21.0	8.33	8	12	1924	150	0.02628	5.9	8.2	4.5	35	1.780	0.190	0.081	0	0.72	0	0.00398	0.000018	0.0102	0.0058	0.023	0.00021	0.00008	2
2015-6-9	14	20.7	8.32	10	16	1764	140	0.01449	6.0	6.1	2.4	39	1.020	0.056	0.055	0	0.80	0	0.00397	0.000013	0.0103	0.0049	0.020	0.00000	0.00000	2
2015-6-9	15	20.4	8.26	6	17	2612	160	0.02434	5.6	6.0	2.4	38	1.750	0.104	0.028	0	0.66	0	0.00306	0.000015	0.0025	0.0050	0.011	0.00019	0.00000	2
2015-6-9	16	20.5	8.28	14	11	1888	160	0.02114	7.1	4.9	4.1	46	3.450	0.304	0.047	0	0.76	0	0.00429	0.000016	0.0156	0.0178	0.020	0.00028	0.00000	2
2015-6-9	17	21.0	8.28	5	12	2988	190	0.00120	5.5	6.6	4.1	47	2.160	0.185	0.032	0	0.77	0	0.00260	0.000012	0.0214	0.0216	0.015	0.00043	0.00000	20
2015-6-9	18	21.0	8.36	8	14	1836	90	0.01636	7.7	9.3	2.4	36	0.984	0.054	0.017	0	0.72	0	0.00573	0.000012	0.0094	0.0028	0.010	0.00014	0.00000	2
2015-6-9	19	22.0	8.60	7	17	1744	100	0.01105	7.6	8.8	4.5	55	1.270	0.065	0.036	0	0.74	0	0.00395	0.000015	0.0050	0.0045	0.000	0.00000	0.00000	2
2015-7-7	1	25.7	8.27	11	32	2344	10	0.01885	7.3	8.3	4.3	63	1.390	0.187	0.047	0.020	0.35	0.092	0.00239	0.000039	0.0017	0.0013	0.040	0.00014	0.00000	170
2015-7-7	2	26.8	7.92	7	19.5	4726	60	0.00932	7.6	8.8	11.5	113	1.970	0.291	0.119	0.031	0.34	0.097	0.00186	0.000044	0.0014	0.0013	0.070	0.00013	0.00026	70
2015-7-7	3	24.5	8.54	8	64	1492	40	0.01243	6.5	7.4	5.2	71	3.180	0.195	0.121	0	0.45	0.155	0.00234	0.000037	0.0019	0.0018	0.043	0.00000	0.00000	110
2015-7-7	4	25.3	8.32	10	20	2412	60	0.01147	6.8	9.7	5.6	49	2.710	0.217	0.042	0.035	0.53	0.087	0.00934	0.000042	0.0027	0.0077	0.069	0.00035	0.00032	260
2015-7-7	5	23.2	8.41	8	10	1845	110	0.00942	7.0	6.2	6.0	36	1.130	0.124	0.037	0	0.41	0	0.00826	0.000059	0.0031	0.0081	0.051	0.00000	0.00000	20

2015-7-7	6	22.1	8.06	3	7	1968	160	0.00800	6.6	7.9	2.4	32	1.370	0.187	0.036	0	0.35	0	0.00278	0.000041	0.0013	0.0000	0.011	0.00000	0.00025	2
2015-7-7	7	27.2	8.57	19	18	2116	140	0.03361	6.2	7.5	2.8	44	1.090	0.123	0.077	0	0.51	0	0.00340	0.000041	0.0018	0.0010	0.025	0.00000	0.00023	70
2015-7-7	8	22.9	8.62	17	24	1984	90	0.02381	6.0	6.9	3.9	37	1.060	0.123	0.104	0	0.53	0	0.00261	0.000043	0.0016	0.0020	0.045	0.00014	0.00000	50
2015-7-7	9	22.3	8.75	20	25	1596	95	0.03226	6.5	11.3	3.0	43	1.000	0.107	0.057	0	0.51	0	0.00351	0.000044	0.0016	0.0017	0.015	0.00010	0.00000	2
2015-7-7	10	25.4	8.61	14	10	2380	62	0.01247	6.1	6.7	2.7	38	2.010	0.299	0.108	0	0.49	0	0.00280	0.000042	0.0019	0.0030	0.018	0.00013	0.00000	2
2015-7-7	11	24.1	8.88	4	7	2052	125	0.01059	4.5	6.4	5.0	69	1.320	0.171	0.085	0	0.47	0	0.00358	0.000041	0.0018	0.0011	0.115	0.00029	0.00000	20
2015-7-7	12	23.7	8.92	20	10	2032	120	0.02641	7.0	8.3	5.0	77	0.989	0.099	0.066	0	0.47	0	0.00366	0.000042	0.0013	0.0000	0.015	0.00000	0.00018	20
2015-7-7	13	25.2	8.54	12	9	2080	145	0.02655	6.8	8.0	4.1	35	0.973	0.091	0.085	0	0.47	0	0.00271	0.000044	0.0016	0.0000	0.049	0.00000	0.00045	2
2015-7-7	14	25.3	8.85	3	4	2008	150	0.01542	7.3	9.6	2.6	36	1.950	0.302	0.028	0	0.45	0	0.00192	0.000028	0.0015	0.0014	0.031	0.00000	0.00300	2
2015-7-7	15	25.0	8.21	20	13	2480	190	0.02459	6.8	10.0	2.3	34	1.850	0.251	0.021	0	0.42	0.000	0.00292	0.000038	0.0017	0.0012	0.019	0.00000	0.00034	2
2015-7-7	16	24.7	8.59	17	14	1988	46	0.02135	6.8	5.4	4.0	44	1.010	0.091	0.115	0	0.51	0	0.00223	0.000041	0.0018	0.0017	0.045	0.00000	0.00011	2
2015-7-7	17	26.4	8.33	15	22	2572	180	0.00121	5.3	10.8	4.0	47	1.480	0.195	0.036	0	0.57	0	0.00279	0.000042	0.0019	0.0000	0.036	0.00038	0.00020	20
2015-7-7	18	26.1	8.51	9	13	2220	80	0.01653	6.0	8.3	2.5	37	2.390	0.307	0.043	0	0.53	0	0.00274	0.000042	0.0018	0.0016	0.077	0.00013	0.00036	2
2015-7-7	19	25.7	8.40	8	32	2104	95	0.01116	5.8	8.2	5.2	72	1.430	0.187	0.028	0	0.61	0	0.00282	0.000042	0.0020	0.0015	0.054	0.00096	0.00039	2
2015-8-4	1	25.0	8.51	15	12	1340	40	0.00758	6.7	8.9	2.6	36	1.210	0.116	0.062	0.021	0.36	0.081	0.00334	0.000009	0.0039	0.0020	0.082	0.00013	0.00160	20
2015-8-4	2	25.7	8.05	4	10	5426	30	0.00519	7.2	9.4	5.2	57	1.790	0.223	0.040	0.023	0.40	0.083	0.00306	0.000044	0.0000	0.0014	0.348	0.00054	0.00031	2
2015-8-4	3	25.0	8.15	11	52	1624	25	0.00219	7.4	6.7	2.1	25	3.760	0.078	0.108	0	0.37	0.123	0.00203	0.000011	0.0011	0.0012	0.072	0.00021	0.00035	80
2015-8-4	4	26.0	8.20	28	28	2400	50	0.01936	5.4	11.6	5.4	61	1.360	0.241	0.062	0	0.60	0.078	0.00325	0.000010	0.0000	0.0012	0.281	0.00053	0.00055	20
2015-8-5	5	25.0	8.40	7	8	852	120	0.00583	5.7	5.4	2.0	18	1.100	0.078	0.062	0	0.31	0	0.00176	0.000011	0.0043	0.0014	0.000	0.00000	0.00027	2
2015-8-5	6	25.0	8.48	4	5	964	140	0.00133	7.1	7.7	2.2	30	1.400	0.078	0.049	0	0.43	0	0.00211	0.000010	0.0000	0.0026	0.000	0.00000	0.00015	2
2015-8-5	7	25.0	8.77	17	15	1112	65	0.00701	6.2	8.9	2.7	37	1.120	0.241	0.085	0	0.37	0	0.00455	0.000012	0.0436	0.0041	0.020	0.00064	0.00010	20
2015-8-5	8	24.0	8.66	24	10	1860	45	0.01177	5.4	7.5	3.3	47	1.050	0.133	0.108	0	0.55	0	0.00394	0.000010	0.0000	0.0000	0.000	0.00049	0.00043	50
2015-8-5	9	24.0	8.68	24	21	1424	50	0.01275	7.3	11.6	3.2	44	1.020	0.126	0.062	0	0.41	0	0.00341	0.000014	0.0011	0.0013	0.010	0.00061	0.00024	2
2015-8-5	10	23.0	8.63	15	9	2216	80	0.01251	6.3	8.8	2.9	41	1.930	0.128	0.085	0	0.52	0	0.00758	0.000013	0.0036	0.0036	0.036	0.00000	0.00047	2
2015-8-5	11	23.0	8.93	17	5	2212	60	0.00746	6.0	10.1	3.3	46	1.410	0.133	0.085	0	0.53	0	0.00470	0.000014	0.0018	0.0000	0.010	0.00044	0.00013	50
2015-8-5	12	24.0	8.76	27	29	1994	40	0.01258	6.6	11.3	3.4	48	1.000	0.080	0.062	0.036	0.48	0	0.00273	0.000020	0.0021	0.0000	0.006	0.00051	0.00020	110
2015-8-5	13	23.5	8.21	20	18	1852	60	0.01737	3.8	10.9	3.1	43	0.992	0.128	0.074	0	0.52	0.054	0.00388	0.000024	0.0040	0.0080	0.019	0.00025	0.00034	2

2015-8-5	14	22.0	7.89	33	17	2160	44	0.02130	4.2	10.5	3.4	47	1.910	0.089	0.112	0	0.57	0	0.00399	0.000015	0.0030	0.0023	0.010	0.00034	0.00021	2
2015-8-5	15	22	8.16	25	18	2328	40	0.01857	4.1	10.9	3.5	48	2.020	0.226	0.074	0	0.62	0	0.00372	0.000022	0.0021	0.0014	0.006	0.00036	0.00030	2
2015-8-5	16	22.0	8.22	25	15	2384	42	0.02144	5.2	8.3	5.4	64	1.150	0.116	0.096	0	0.62	0	0.00406	0.000015	0.0016	0.0028	0.007	0.00044	0.00023	2
2015-8-5	17	24.0	8.10	5	86	3124	95	0.01285	8.1	10.5	11.5	94	1.500	0.218	0.037	0	1.09	0	0.00226	0.000044	0.0046	0.0067	0.029	0.00017	0.00054	2
2015-8-5	18	25.0	8.45	25	28	2052	25	0.00893	5.4	7.9	3.0	41	2.570	0.241	0.085	0	0.53	0	0.00427	0.000014	0.0011	0.0019	0.000	0.00013	0.00034	20
2015-8-5	19	22.0	7.93	15	13	1976	80	0.00804	3.2	7.6	2.9	41	1.590	0.089	0.110	0	0.55	0	0.00357	0.000027	0.0054	0.0000	0.005	0.00021	0.00057	70
2015-9-1	1	25.0	8.69	16	19	1408	45	0.02737	5.6	9.2	2.6	36	1.010	0.080	0.161	0.025	0.59	0.078	0.00133	0.000023	0.0059	0.0097	0.000	0.00000	0.00000	170
2015-9-1	2	25.0	8.10	4	13	4948	48	0.00221	7.2	7.9	5.0	58	1.320	0.146	0.024	0.026	0.49	0.092	0.00124	0.000028	0.0028	0.0099	0.008	0.00000	0.00000	230
2015-9-1	3	25.0	8.47	31	51	1656	10	0.01000	7.6	5.9	2.0	41	2.910	0.183	0.134	0	0.57	0.117	0.00102	0.000026	0.0020	0.0017	0.000	0.00016	0.00000	260
2015-9-1	4	25.0	8.28	26	28	2684	45	0.01453	3.5	11.3	4.1	48	1.200	0.223	0.058	0	0.84	0.080	0.00128	0.000030	0.0017	0.0000	0.033	0.00000	0.00000	110
2015-9-1	5	23.9	9.31	3	8	940	135	0.00313	5.1	5.4	2.0	19	1.040	0.135	0.036	0	0.47	0	0.00094	0.000027	0.0066	0.0142	0.000	0.00012	0.00000	50
2015-9-1	6	23.9	8.27	3	4	904	120	0.01056	7.8	6.2	2.0	26	1.300	0.215	0.047	0	0.59	0	0.00095	0.000050	0.0030	0.0084	0.000	0.00026	0.00000	2
2015-9-1	7	23.0	8.96	19	14	1244	82	0.01200	3.9	9.5	4.1	69	1.030	0.135	0.077	0	0.54	0	0.00095	0.000032	0.0041	0.0054	0.000	0.00000	0.00000	2
2015-9-1	8	22.3	8.96	21	17	1400	60	0.02356	4.1	11.1	3.9	61	1.090	0.148	0.124	0	0.59	0	0.00113	0.000030	0.0038	0.0058	0.006	0.00016	0.00000	2
2015-9-1	9	21.9	8.76	15	19	2040	50	0.01056	4.9	12.3	3.0	41	1.110	0.148	0.096	0	0.70	0	0.00169	0.000030	0.0040	0.0109	0.000	0.00000	0.00000	2
2015-9-1	10	25.7	8.98	5	5	1514	120	0.00469	3.6	7.2	2.1	25	1.780	0.080	0.047	0	0.55	0	0.00153	0.000033	0.0033	0.0129	0.000	0.00019	0.00000	2
2015-9-1	11	25.1	8.95	11	11	2016	110	0.00278	3.5	10.6	2.9	41	1.060	0.240	0.077	0	0.55	0	0.00196	0.000032	0.0033	0.0060	0.009	0.00000	0.00011	20
2015-9-1	12	21.0	8.93	22	33	1748	70	0.01285	3.5	11.2	3.5	73	1.100	0.125	0.081	0.022	0.59	0	0.00167	0.000037	0.0133	0.0125	0.017	0.00014	0.00000	20
2015-9-1	13	25.3	8.91	9	11	1704	100	0.00439	3.7	9.8	3.5	55	1.020	0.125	0.047	0	0.53	0	0.00186	0.000036	0.0038	0.0087	0.008	0.00018	0.00022	50
2015-9-1	14	24.2	8.70	14	13	2500	90	0.00712	4.9	9.8	3.6	56	1.820	0.352	0.062	0	0.65	0	0.00145	0.000036	0.0039	0.0100	0.000	0.00031	0.00000	20
2015-9-1	15	26.7	8.42	22	15	2504	50	0.00930	3.7	11.1	5.0	56	2.100	0.134	0.070	0	0.78	0	0.00094	0.000054	0.0045	0.0053	0.020	0.00020	0.00000	2
2015-9-1	16	27.0	8.46	17	18	2324	60	0.01206	4.6	12.1	3.8	51	1.210	0.233	0.096	0	0.76	0	0.00152	0.000039	0.0020	0.0036	0.000	0.00028	0.00042	20
2015-9-1	17	24.7	8.23	23	14	2396	50	0.00883	3.1	11.6	5.5	55	1.610	0.161	0.070	0	0.86	0	0.00128	0.000051	0.0036	0.0095	0.011	0.00032	0.00000	2
2015-9-1	18	25.8	8.76	67	38	1956	20	0.00780	4.9	9.3	4.0	61	2.320	0.146	0.059	0	0.65	0	0.00159	0.000042	0.0032	0.0026	0.006	0.00000	0.00000	2
2015-9-1	19	24.1	8.50	43	42	1990	40	0.00530	2.9	10.5	4.1	67	1.590	0.195	0.035	0	0.73	0	0.00162	0.000043	0.0033	0.0055	0.046	0.00029	0.00047	2
2015-10-8	1	13.0	8.42	41	16	2016	50	0.12386	8.3	7.1	4.1	38	1.940	0.238	0.221	0.025	0.57	0.079	0.00292	0.000029	0.0024	0.0193	0.041	0.00000	0.00012	140
2015-10-8	2	12.2	8.51	13	12	1544	60	0.02778	7.8	5.5	3.0	27	4.760	1.670	0.081	0.021	0.49	0.085	0.00099	0.000028	0.0017	0.0026	0.015	0.00000	0.00053	170

2015-10-8	3	12.0	8.69	32	101	768	20	0.00614	7.2	5.0	2.7	32	1.970	0.341	0.222	0	0.43	0.120	0.00092	0.000030	0.0022	0.0020	0.012	0.00000	0.00017	80
2015-10-8	4	12.2	8.33	29	22	2208	40	0.03377	2.9	12.1	4.5	47	1.330	0.154	0.065	0	0.81	0.077	0.00286	0.000029	0.0022	0.0000	0.013	0.00031	0.00049	70
2015-10-8	5	11.9	8.27	10	10	728	120	0.02113	6.0	4.7	2.1	16	1.950	0.238	0.048	0	0.45	0	0.00076	0.000031	0.0011	0.0036	0.006	0.00000	0.00020	50
2015-10-8	6	11.8	8.45	12	6	696	70	0.01340	5.8	3.8	2.1	17	1.150	0.079	0.058	0	0.44	0	0.00095	0.000028	0.0022	0.0039	0.012	0.00000	0.00032	2
2015-10-8	7	12.5	8.36	10	16	764	105	0.02954	5.4	4.2	2.4	25	1.900	0.238	0.081	0	0.47	0	0.00123	0.000031	0.0012	0.0011	0.011	0.00000	0.00021	2
2015-10-8	8	12.3	8.66	13	12	960	90	0.03470	6.7	8.1	2.4	35	1.100	0.079	0.192	0	0.53	0	0.00153	0.000028	0.0020	0.0144	0.043	0.00028	0.00034	2
2015-10-8	9	12.0	8.70	11	20	1380	110	0.02464	5.9	9.7	2.8	34	1.230	0.144	0.100	0	0.53	0	0.00274	0.000032	0.0017	0.0010	0.009	0.00013	0.00035	2
2015-10-8	10	12.3	8.78	16	6	940	70	0.06070	5.9	6.0	2.4	31	2.740	0.417	0.069	0	0.46	0	0.00197	0.000031	0.0014	0.0000	0.015	0.00000	0.00011	2
2015-10-8	11	12.4	8.76	14	12	1072	110	0.03235	4.3	7.3	2.2	21	1.520	0.135	0.085	0	0.46	0	0.00155	0.000032	0.0023	0.0011	0.016	0.00000	0.00030	20
2015-10-8	12	12.6	8.52	17	32	1208	120	0.04047	6.5	7.7	2.6	28	1.840	0.229	0.088	0	0.59	0	0.00220	0.000033	0.0017	0.0026	0.022	0.00024	0.00094	50
2015-10-8	13	12.2	8.43	11	10	1260	70	0.03265	5.3	6.6	2.5	30	2.570	0.388	0.050	0	0.57	0	0.00237	0.000033	0.0019	0.0015	0.028	0.00016	0.00010	70
2015-10-8	14	12.5	8.60	21	14	1232	50	0.03566	5.8	10.5	2.3	35	1.420	0.126	0.073	0	0.61	0	0.00229	0.000036	0.0015	0.0011	0.011	0.00000	0.00000	110
2015-10-8	15	13.2	8.28	21	16	2088	70	0.03902	5.0	10.9	2.4	39	2.010	0.238	0.073	0	0.78	0	0.00218	0.000036	0.0025	0.0000	0.016	0.00026	0.00000	2
2015-10-8	16	12.5	8.24	14	20	1960	90	0.02617	5.5	9.7	2.8	53	1.020	0.088	0.119	0	0.75	0	0.00221	0.000035	0.0017	0.0044	0.016	0.00014	0.00013	20
2015-10-8	17	12.7	8.33	24	18	2108	50	0.02605	5.2	9.1	3.6	51	1.550	0.135	0.069	0	0.81	0	0.00247	0.000037	0.0020	0.0015	0.030	0.00013	0.00070	2
2015-10-8	18	12.8	8.39	8	34	1176	130	0.01740	5.2	6.4	2.3	26	1.920	0.238	0.066	0	0.60	0	0.00135	0.000040	0.0015	0.0025	0.019	0.00000	0.00000	130
2015-10-8	19	10.0	8.44	8	41	1488	120	0.01782	4.5	7.0	2.7	35	1.360	0.154	0.054	0	0.68	0	0.00195	0.000042	0.0016	0.0011	0.024	0.00018	0.00022	50
2015-11-3	1	8.2	8.19	8	11	2344	30	0.01720	4.7	7.9	2.1	19	1.35	0.080	0.081	0	0.55	0.087	0.00128	0.000056	0.0018	0.0000	0.229	0.00028	0.00000	220
2015-11-3	2	7.9	8.11	6	19	3256	40	0.00850	4.0	8.5	2.6	23	1.40	1.400	0.074	0.021	0.53	0.093	0.00130	0.000054	0.0016	0.0000	0.183	0.00030	0.00000	330
2015-11-3	3	11.4	8.07	16	16	1804	60	0.00340	7.9	5.4	4.1	46	5.540	0.243	0.165	0	0.53	0.105	0.00158	0.000059	0.0018	0.0024	0.147	0.00028	0.00000	80
2015-11-3	4	9.3	8.21	5	8	1744	110	0.00270	5.7	7.7	4.5	44	1.260	0.161	0.081	0	0.63	0.080	0.00157	0.000039	0.0021	0.0011	0.020	0.00033	0.00016	80
2015-11-3	5	6.7	8.13	3	13	1684	120	0.00154	5.8	4.3	2.1	19	1.050	0.080	0.096	0	0.47	0	0.00129	0.000050	0.0023	0.0029	0.027	0.00056	0.00000	20
2015-11-3	6	6.1	8.28	10	9	1616	80	0.00966	6.1	5.4	2.2	21	3.410	0.068	0.089	0	0.47	0	0.00129	0.000049	0.0000	0.0013	0.035	0.00028	0.00000	2
2015-11-3	7	6.3	8.02	3	9	1084	110	0.00679	4.0	5.0	2.4	22	1.920	0.076	0.123	0	0.44	0	0.00103	0.000072	0.0018	0.0015	0.019	0.00039	0.00000	3
2015-11-3	8	6.4	8.15	8	14	1166	110	0.00450	5.0	8.61	3.5	31	2.100	0.137	0.104	0	0.53	0	0.00212	0.000045	0.0018	0.0010	0.074	0.00029	0.00000	4
2015-11-3	9	6.2	8.10	3	8	1300	130	0.00402	5.0	5.9	2.6	26	1.650	0.073	0.112	0	0.55	0	0.00161	0.000047	0.0025	0.0020	0.018	0.00047	0.00000	5
2015-11-3	10	7.3	8.27	12	10	1256	90	0.00747	4.7	6.2	2.2	21	1.800	0.260	0.092	0	0.47	0	0.00146	0.000039	0.0061	0.0014	0.044	0.00047	0.00000	2

2015-11-3	11	7.4	8.29	7	8	1044	110	0.00729	7.8	6.1	2.1	23	1.680	0.030	0.089	0	0.46	0	0.00130	0.000041	0.0015	0.0000	0.031	0.00036	0.00000	20
2015-11-3	12	6.4	8.80	9	10	1084	80	0.00428	3.7	8.4	2.1	21	2.010	0.457	0.081	0	0.53	0	0.00119	0.000042	0.0016	0.0000	0.020	0.00035	0.00000	80
2015-11-3	13	7.3	8.31	3	5	1068	120	0.00360	3.9	6.5	2.5	24	1.590	0.030	0.081	0	0.49	0	0.00117	0.000041	0.0015	0.0000	0.065	0.00027	0.00000	40
2015-11-3	14	7.2	8.42	4	13	1200	120	0.00249	5.0	8.7	2.9	30	1.900	0.080	0.112	0	0.57	0	0.00141	0.000050	0.0015	0.0000	0.029	0.00025	0.00016	2
2015-11-3	15	7.1	8.30	7	14	1580	130	0.00429	5.9	8.2	2.8	29	1.390	0.170	0.074	0	0.63	0	0.00154	0.000043	0.0072	0.0000	0.096	0.00028	0.00000	2
2015-11-3	16	7.1	8.12	3	5	1336	120	0.00437	5.1	6.6	2.6	25	1.500	0.063	0.081	0	0.61	0	0.00140	0.000051	0.0045	0.0000	0.145	0.00050	0.00000	20
2015-11-3	17	7.5	8.28	7	11	1132	120	0.00439	5.9	7.9	3.1	32	1.160	0.202	0.138	0	0.68	0	0.00169	0.000051	0.0019	0.0000	0.023	0.00025	0.00000	20
2015-11-3	18	7.4	8.17	3	8	1132	110	0.00333	5.1	6.4	2.5	23	1.010	0.260	0.089	0	0.87	0	0.00108	0.000042	0.0017	0.0000	0.026	0.00035	0.00000	170
2015-11-3	19	7.1	8.34	8	17	1142	110	0.00316	6.0	6.3	2.2	28	1.240	0.202	0.074	0	0.46	0	0.00149	0.000043	0.0015	0.0000	0.018	0.00029	0.00000	50

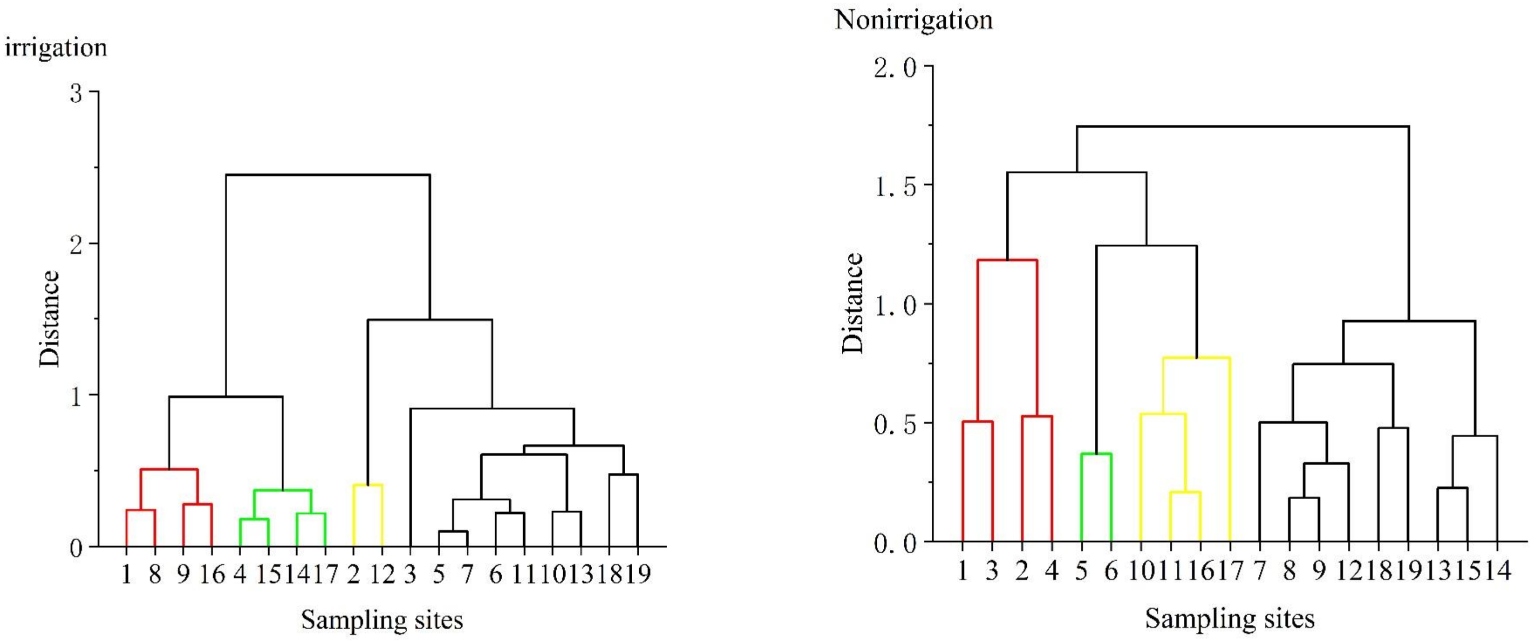

Figure S1 CA dendrograms for the irrigation and non-irrgation periods.
